# Prokaryotic diversity and biogeochemical characteristics of benthic microbial ecosystems at La Brava, a hypersaline lake at Salar de Atacama, Chile

**DOI:** 10.1371/journal.pone.0186867

**Published:** 2017-11-15

**Authors:** Maria Eugenia Farias, Maria Cecilia Rasuk, Kimberley L. Gallagher, Manuel Contreras, Daniel Kurth, Ana Beatriz Fernandez, Daniel Poiré, Fernando Novoa, Pieter T. Visscher

**Affiliations:** 1 Laboratorio de Investigaciones Microbiológicas de Lagunas Andinas (LIMLA), Planta Piloto de Procesos Industriales Microbiológicos (PROIMI), CCT-Tucumán, CONICET, Tucumán, Argentina; 2 Centro de Ecología Aplicada (CEA), Ñuñoa, Santiago, Chile; 3 Centro de Investigaciones Geológicas, Universidad Nacional de La Plata-Conicet, La Plata, Argentina; 4 Department of Marine Sciences, University of Connecticut, Groton, Connecticut, United States of America; 5 Australian Centre for Astrobiology, University of New South Wales, Sydney, New South Wales, Australia; University of Illinois at Chicago, UNITED STATES

## Abstract

Benthic microbial ecosystems of Laguna La Brava, Salar de Atacama, a high altitude hypersaline lake, were characterized in terms of bacterial and archaeal diversity, biogeochemistry, (including O_2_ and sulfide depth profiles and mineralogy), and physicochemical characteristics. La Brava is one of several lakes in the Salar de Atacama where microbial communities are growing in extreme conditions, including high salinity, high solar insolation, and high levels of metals such as lithium, arsenic, magnesium, and calcium. Evaporation creates hypersaline conditions in these lakes and mineral precipitation is a characteristic geomicrobiological feature of these benthic ecosystems. In this study, the La Brava non-lithifying microbial mats, microbialites, and rhizome-associated concretions were compared to each other and their diversity was related to their environmental conditions. All the ecosystems revealed an unusual community where *Euryarchaeota*, *Crenarchaeota*, *Acetothermia*, *Firmicutes* and *Planctomycetes* were the most abundant groups, and cyanobacteria, typically an important primary producer in microbial mats, were relatively insignificant or absent. This suggests that other microorganisms, and possibly novel pathways unique to this system, are responsible for carbon fixation. Depth profiles of O_2_ and sulfide showed active production and respiration. The mineralogy composition was calcium carbonate (as aragonite) and increased from mats to microbialites and rhizome-associated concretions. Halite was also present. Further analyses were performed on representative microbial mats and microbialites by layer. Different taxonomic compositions were observed in the upper layers, with *Archaea* dominating the non-lithifying mat, and *Planctomycetes* the microbialite. The bottom layers were similar, with *Euryarchaeota*, *Crenarchaeota* and *Planctomycetes* as dominant phyla. Sequences related to *Cyanobacteria* were very scarce. These systems may contain previously uncharacterized community metabolisms, some of which may be contributing to net mineral precipitation. Further work on these sites might reveal novel organisms and metabolisms of biotechnological interest.

## Introduction

The role of microorganisms in geological processes, particularly in microbially-induced mineral precipitation has gained much attention recently [1–6]. Microbes alter the geochemistry of their immediate microenvironment through metabolic activities and, as such, have the potential to induce or influence mineral precipitation or dissolution by affecting the saturation index of the specific mineral [7–11]. In addition, exopolymeric substances (EPS), which are produced by several mat organisms [12, 13], play a key role. The organic matrix of EPS contains anionic functional groups capable of binding metal ions like Ca^2+^. The bound ions may function as nucleation sites, or in the case of calcium carbonate, mineral precipitation may result from liberation of bound Ca^2+^ and dissolved inorganic carbon when microbial and physicochemical degradation occurs [2, 13, 14]. This organomineralization process can result in the lithification of microbial mats, forming microbialites [1].

Actively forming mats and microbialites are present in habitats with a range of environmental conditions, typically where predators and burrowing eukaryotes are in low abundance. Examples include hypersaline systems such as Hamelin Pool in Shark Bay, Western Australia [15, 16], the solar salterns of Guerrero Negro in Mexico [17], the hypersaline lakes on Eleuthera, Bahamas [2], and hypersaline lagoons in coastal Brazil [18]. Other extreme examples include hot springs such as Obsidian Pool in Yellowstone National Park [19], Shionoha in Japan [20, 21] or Frying Pan Lake in New Zealand [22]. Mats and microbialites also occur in less extreme environments such as the open marine stromatolites and thrombolites of Exuma Sound in the Bahamas [23, 24], freshwater microbialites at the Cuatro Ciénagas Basin in Mexico [25, 26], Ruidera Pools in Spain [27], and Pavilion Lake in British Columbia, Canada [28].

Recently, a range of lithifying microbial ecosystems, have been described in hypersaline lakes in the Andean plateau. These include aragonitic microbialites in Laguna Socompa, Argentina (3600 masl) [29], aragonitic oncolites in Laguna Negra, Argentina (4600 masl) [30], biofilms with carbonate minerals, notably gaylussite, in Laguna Diamante, Argentina (4600 masl) [31], microbialites comprised of halite-aragonite and aragonite-calcite at La Brava, Chile (2300 masl) [32], gypsum endoevaporites at Llamara, Chile, Laguna de Piedra, Chile (2340 masl) and Tebenquiche, Chile (2500 masl) [32–37].

A previous survey of Laguna La Brava and Laguna Tebenquiche, both shallow hypersaline lakes in the southwest of Salar de Atacama, revealed a variety microbial ecosystems ranging from non-lithifying mats to flat and domal microbialites [32]. In that investigation, the diversity study focused on *Bacteria*, excluding the domain *Archaea*. Recently, a study in Lake Tebenquiche microbial ecosystems revealed that Archaea comprised most of the microbial diversity and included phyla capable of methanogenesis [35]. Here, we similarly expanded the knowledge of La Brava´s diversity by using primers which amplify the V4 variable region of both Archaea and Bacteria, and by increasing the variety of ecosystems studied including one microbial mat, two microbialites, and one rhizome-associated concretion. We also related these systems to the geochemistry of their immediate environments, and measured the oxygen and sulfide concentrations in their porewater using microelectrodes. Further analysis of prokaryotic diversity by layer in two representative ecosystems was also included.

## Materials and methods

### Site description

Salar de Atacama is a distinct geomorphologic structure in the north of Chile [38] and is the oldest and the largest evaporitic basin in that country. It is a tectonic intramontane basin filled with Tertiary to Quaternary clastic and evaporitic sediments of continental origin. The hydrogeological setting of the Salar is quite complex, mostly receiving an input of groundwater input and some surface water, predominantly from the east [39]. The dominant input is due to upwelling of groundwater containing leached volcanic material. In the lowest region of Atacama basin, groundwater surfaces creating a series of lakes including Laguna de Piedra, Tebenquiche, Chaxas, Burro Muerto and La Brava [40]. Laguna La Brava is a shallow hypersaline lake surrounded by the Salar’s gypsum crust. During summer, the water level falls because of the evaporation exceeding water input, increasing the salinity [32]. Most of the shoreline is covered by a continuous microbial mat, part of which desiccates during the dry season. The accumulation of gases underneath the leathery surface results in the formation of different globe-shaped structures with small domal, cerebroid, snake-like morphologies [32]. The environmental conditions in which these lakes form are characterized by (1) high solar radiation, including UV [41] due to less light scatter, (2) extreme diel temperature fluctuations typical of desert environments, (3) net evaporation producing hypersaline water [40], (4) extremely low relative humidity [42] and (5) high arsenic and lithium concentrations in the water due to volcanic events [43].

### Sample collection

**Fig 1:**
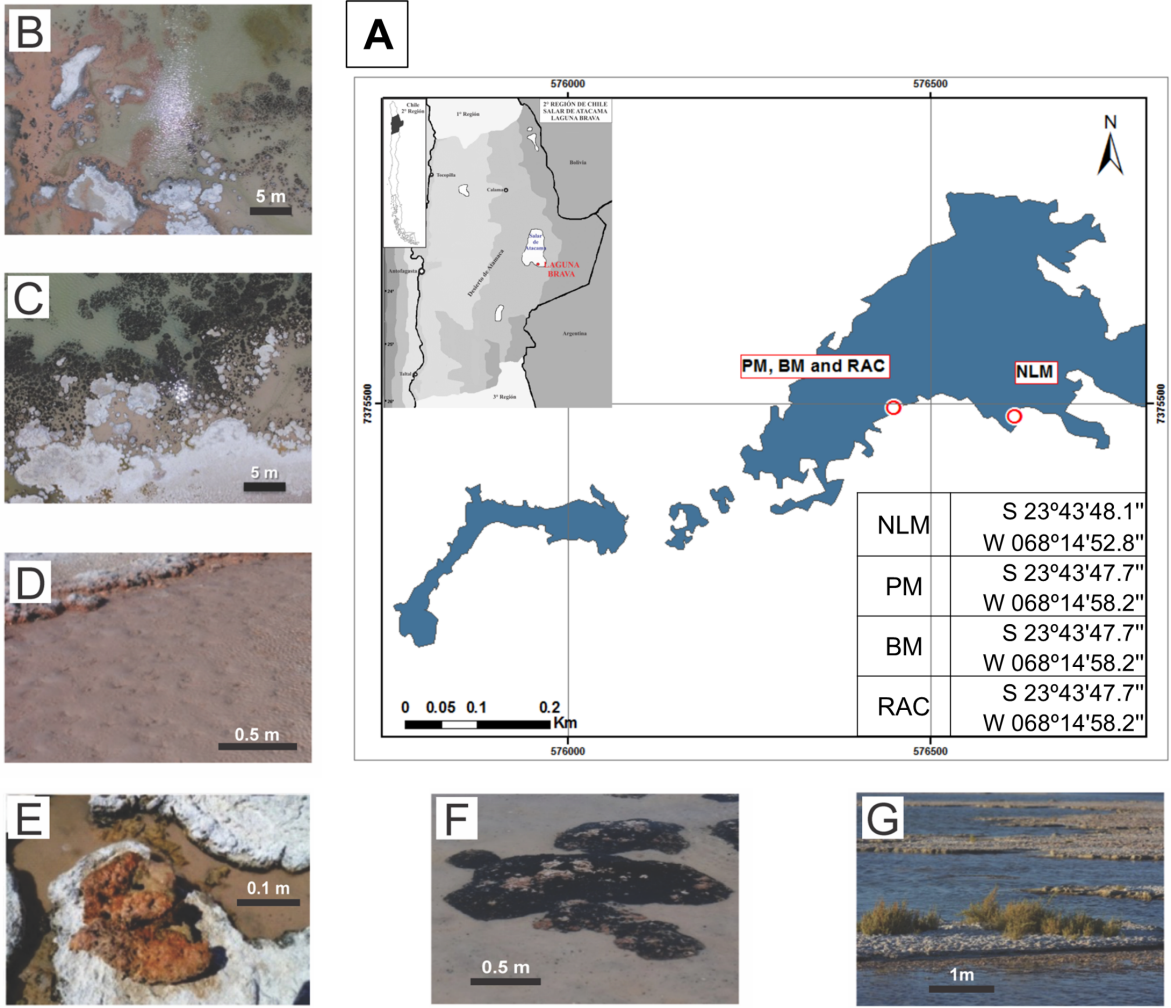
Site location and images showing systems studied. **(**A) Aerial view of Laguna La Brava indicating the sampling sites. (B) Aerial view of NLM (scale bar 5m). (C) Aerial view of microbialite site. (D) Detail of B, showing NLM (scale bar 0.5m). (E) Top view showing detail of pink mat (PM; scale bar 0.1m). (F) View of black mat (BM, scale bar 0.5m). (G) View from the side of *Distichlis spicata* (*Gramineae*), with underground rhizome-associated concretions not visible, scale bar 1m).

Four sediment ecosystem types were collected from the south side of the Laguna La Brava (Fig 1A). This location is freely accessible, with no protected areas from Chilean government, and no specific permissions were required for these activities.

The site of the non-lithifying mat (NLM) was located on the west of a water input channel with the lowest conductivity (98 mS.cm^-1^) and highest turbidity (10.30 NTU) of all sites sampled. The NLM surface consisted of a continuous pink layer covered by 3–5 cm of water during the dry season and 5–10 cm during the wet season (Fig 1B and 1D). Microbialites (lithifying) are most abundant along the southwestern shoreline (Fig 1C) and have semi-spheroidal morphologies covered by pink or black leathery biofilms (PM and BM, respectively). They grow upward until they reach the water/air interface after which some expand laterally forming horizontal beds. Two kinds of submerged microbialites were sampled in this study (November 2012). The first dome, pink microbialite (PM, Fig 1E) was located near the shoreline, covered by a pink biofilm, and appeared to be a softer lithified microbialite than the second dome, black microbialite (BM, Fig 1F) which was covered by a black crust and located toward the middle of the lake, approximately 1.5 meter away. The rhizome-associated concretion (RAC) was sampled from the shore of the lake, where *Distichlis spicata* (*Gramineae*) grows abundantly on a white mineral substrate containing a laminated microbial subsurface community (Fig 1G), which resembles the microbialite beds described above.

For water analyses, overlying water samples for NLM, BM/PM, and water adjacent to plants harboring RAC were collected and stored in acid-cleaned bottles on ice in the dark for analyses in the laboratory within 48 h.

For DNA analyses of all mats and microbialites, triplicate cores (2 cm^2^ each) were taken to a depth of 3 cm and pooled prior to homogenizing in order to obtain representative samples. Further analysis for the depth distribution of diversity was performed on layers of NLM and BM, which were sampled based on their appearance. NLM layers were taken from 0–5 mm (layer 1), 5–10 mm (layer 2), 10–20 mm (layer 3) and 20–30 mm (layer 4) depth horizon. BM layers were taken from 0–3 mm (layer 1), 3–7 mm (layer 2) and 7–12 mm (layer 3). Homogenates used for DNA extraction were stored at -20°C in the dark and processed within a week.

### Analyses

#### Water column

The temperature and pH of the water column were determined *in situ*. Samples were stored in acid-cleaned bottles on ice in the dark until analyses in the laboratory within 48 h. Dissolved oxygen, salinity, conductivity, total P, NO_3_^-^, NO_2_^-^, Ca^2+^, Mg^2+^, K^+^, SO_4_^2−^, and Na^+^, were measured according to the methodology described by [44]. NH_4_^+^, orthophosphate, and total organic nitrogen were analyzed using a Merck Nova 60 Spectro Photometer by following standard methods (American Public Health Association 1998).

#### Sediment geochemistry

Microelectrodes were used to obtain depth profiles of the oxygen and sulfide concentrations *in situ* [32, 45] during the peak of photosynthesis when the intensity of photosynthetically active radiation (PAR) was between 1,410 and 2,550 μE.m^-2^.s^-1^. Oxygen was determined with a Clark-type probe, and sulfide using an amperometric sensor (Unisense, Aarhus, Denmark). Both O_2_ and H_2_S needle probes had internal reference, guard and measuring electrodes and were connected to a modified portable picoammeter (Unisense PA 2000, Aarhus, Denmark). The electrodes were calibrated in the laboratory before and after field measurements. Between measurements in the field, electrodes were checked by a two-point calibration. Three to five replicate profiles covering the upper 10–15 mm of each sample were determined. The oxygen and sulfide concentrations were corrected for altitude according to [32, 46]. PAR (400–700 nm) was measured using a LiCor LI 250A light meter with a LiCor LI-192 underwater quantum sensor. UV A-B (280–400 nm) measurements were made with a Solar Light Co. PMA 2100 radiometer (Solar Light Company, Inc., Glenside, PA, USA).

For additional comparison of sulfur cycling between La Brava and Tebenquiche systems, thiosulfate and polysulfides were measured in porewater samples from La Brava (NLM, PM and BM) and Tebenquiche (MA1 and MA2). These samples were recovered by centrifugation (2 min at 15,000 rpm) of mat plugs. All mats were sampled during peak photosynthesis (noon-2:00pm). Selected mats (NLM, PM and MA1) were also sampled at the end of the night. Samples from each site were obtained by triplicate. Sulfate-sulfur concentrations in polysulfides and thiosulfate were determined colorimetrically after cyanolysis [45, 47]. Aliquots of 100 μL were incubated in a buffered 0.2M cyanide solution for 30 min (pH = 4.8 for thiosulfate at room temperature; pH = 8.7 for polysulfides at 90°C). After incubation and addition of Fe(III), the concentration of the resulting ferrithiocyanide complex was determined by absorption at 560nm. Na_2_S_2_O_3_ and KSCN were used as standards.

Bulk samples for mineral analyses of all microbial sediments were collected in triplicates and kept at 4°C in the dark prior to analysis. The mineral composition was determined by X-ray diffraction (XRD) analysis of air-dried, finely ground (<20 μm) samples of non-lithifying mat, microbialites and rhizome-associated concretion with a PANalyticalX´Pert PRO diffractometer, with Cu lamp (kα = 1.5403 Å) operated at 40 mA and 40 kV at Centro de Investigaciones Geológicas (La Plata, Argentina). Organic content of mat and microbialite samples was measured after drying at 105°C for 24hr followed by heating at 550°C for one hr [48].

#### Sediment DNA

Total genomic DNA was obtained from 0.2 g of material using the protocol supplied in the Power Biofilm DNA Isolation Kit (MO BIO Laboratories, Carlsbad, CA.). The extracted DNA samples were amplified with the RK primers (F515 and R806) [49] targeting the hypervariable V4 region of the prokaryotic 16S rRNA gene. Forward and reverse PCR primers contain a 454 adapter A and B, respectively, and a 10 nucleotide “multiple identifier” (MID). Five reactions via PCR were performed to reduce bias. Reactions (25 μl final volume) consisted of final concentrations of 2.5 μl FastStart High Fidelity 10X Reaction Buffer (Roche Applied Science, Mannheim, Germany), 0.2 mM dNTPs, 20 ng of template DNA, 0.4 μM forward and reverse primers targeting the V4 hypervariable region of the 16S rRNA gene, and 1.25 units FastStart High Fidelity Enzyme Blend (Roche Applied Science). PCR cycling conditions consisted of 95°C for 5 min, followed by 30 cycles of 95°C for 45 s, 57°C for 45 s, and 72°C for 60 s, and a final extension at 72°C for 4 min. The five PCR amplicons were pooled and purified using AMPure XP beads (Agencourt Bioscience, Beckman Coulter, Brea, CA, USA) then analysed with a Quant-IT Pico Green dsDNA Kit (Invitrogen Molecular Probes Inc, Eugene, Oregon, USA). The composition of the purified amplicons of the V4 region of 16S rRNA genes was determined by pyrosequencing using a Roche 454 FLX titanium sequencer (Roche Applied Science).

All results of the pyrosequencing runs were deposited in the NCBI Sequence Read Archive (SRA) database under the accession number SRP063322. Samples were analysed at INDEAR genome sequencing facility (Santa Fe, Argentina). 31,575 filtered sequences with an average length of 253 bp were obtained from 11 samples. Filter parameters were set to reject reads that had mean quality score <25, maximum homopolymer run >6, number of primer mismatches >0, and read length <200 bp or >1000 bp.

#### Taxonomy-based analysis and functional assignment

Diversity of the microbial community was assessed by sequence analysis the of the V4 hypervariable region of bacterial 16S rRNA using the QIIME software package v.1.7.0 [50]. Sequences were clustered into OTUs using UCLUST [51] at the 97% similarity level using the most abundant sequence as the representative sequence for each OTU. A table was compiled with the number of sequences per OTU. Each representative OTU sequence was characterized taxonomically with the RDP classifier [52] based on the Greengenes database version 12.10 [53] using a bootstrap confidence of 50%. OTUs assigned to chloroplasts or mitochondria were removed from the analysis.

Functional assignments were inferred based on literature search. From the taxonomic classification of OTUs, functional groups were attributed according to reported metabolisms for each known taxon [54].

From the OTU table, lists for each sample were obtained, and a Venn graph was generated using jvenn tool [55]. OTU tables were subsampled using 10 replicates for each sampling effort at increasing intervals of 100 sequences. Alpha diversity indices were calculated on each subsample of the rarefaction curve and on the complete OTU table (including all sequences) using QIIME. Alpha diversity metrics calculated included observed species, CHAO1, Shannon, Simpson, Equitability and Dominance indices. Results from bulk samples were compared using the principal coordinate analysis (PCoA) implemented in QIIME. Briefly, OTU tables were rarefied, and weighted unifrac distance matrices were built for each rarefied table. A jackknifed replicate PCoA plot was obtained from all these matrices.

#### Multivariate analysis of water column chemistry and ecosystem types

A Canonical Correspondence Analysis (CCA) was performed to correlate environmental variables with prokaryotic phyla and samples. To assure the significance of all canonical axes was carried out a Monte Carlo test with 499 permutations. We used CANOCO 4.5 software package (Microcomputer Power, Ithaca, NY, USA) to make the CCA and the tool CANODRAW for triplot visualization [56].

## Results

### Water column

The ratio of major ions in the water column was similar in all the sampling sites: Cl^-^> SO_4_^2-^ for anions and Na^+^ > K^+^>Mg^2+^>Ca^2+^ for cations (Table 1). However, the conductivity was the lowest and turbidity was the highest at NLM (98 mS/cm and 10.30 NTU at NLM, 108 mS/cm and 2.69 NTU at the PM and BM sampling area, and 103mS/cm and 2.55 NTU at RAC) (Table 1).

**Table 1:** Water column physicochemical characteristics.

Physico-chemical properties	Unit	NLM	PM/BM	RAC
Salinity	g/L	71	80	75
Hardness	mg/L	9716	14084	12292
pH	-	8.1	8.2	8.1
Total Alkalinity	mg CaCO_3_/L	521	645	593
Temperature	°C	28.8	27.2	23.6
Turbidity	NTU	10.30	2.69	2.55
Nitrate	μg/L	739	1101	1038
Nitrite	μg/L	<0.2	0.6	<0.2
Total Organic Nitrogen	μg/L	1175	1105	1065
Total Phosphorus	ug/L	2525	3200	3075
Orthophosphate	μg/L	2410	3115	2700
Sulfate	mg/L	4890	5626	5341
Sulfur	mg/L	1632	1878	1783
Total Sulfide	mg/L	< 0.2	< 0.2	< 0.2
Magnesium	mg/L	2011	2455	2418
Calcium	mg/L	576	1593	936
Dissolved Arsenic	mg/L	9	11	10
Total Lithium	mg/L	404	443	360
Biochemical oxygen demand (BOD)	mg/L	3.8	3.9	2.8
Chemical oxygen demand (COD)	mg/L	178	218	128
Chlorophyll a	ug/L	1	<0.1	1
Total Organic Matter	mg/L	10	16	20

### Sediment geochemistry

**Fig 2:**
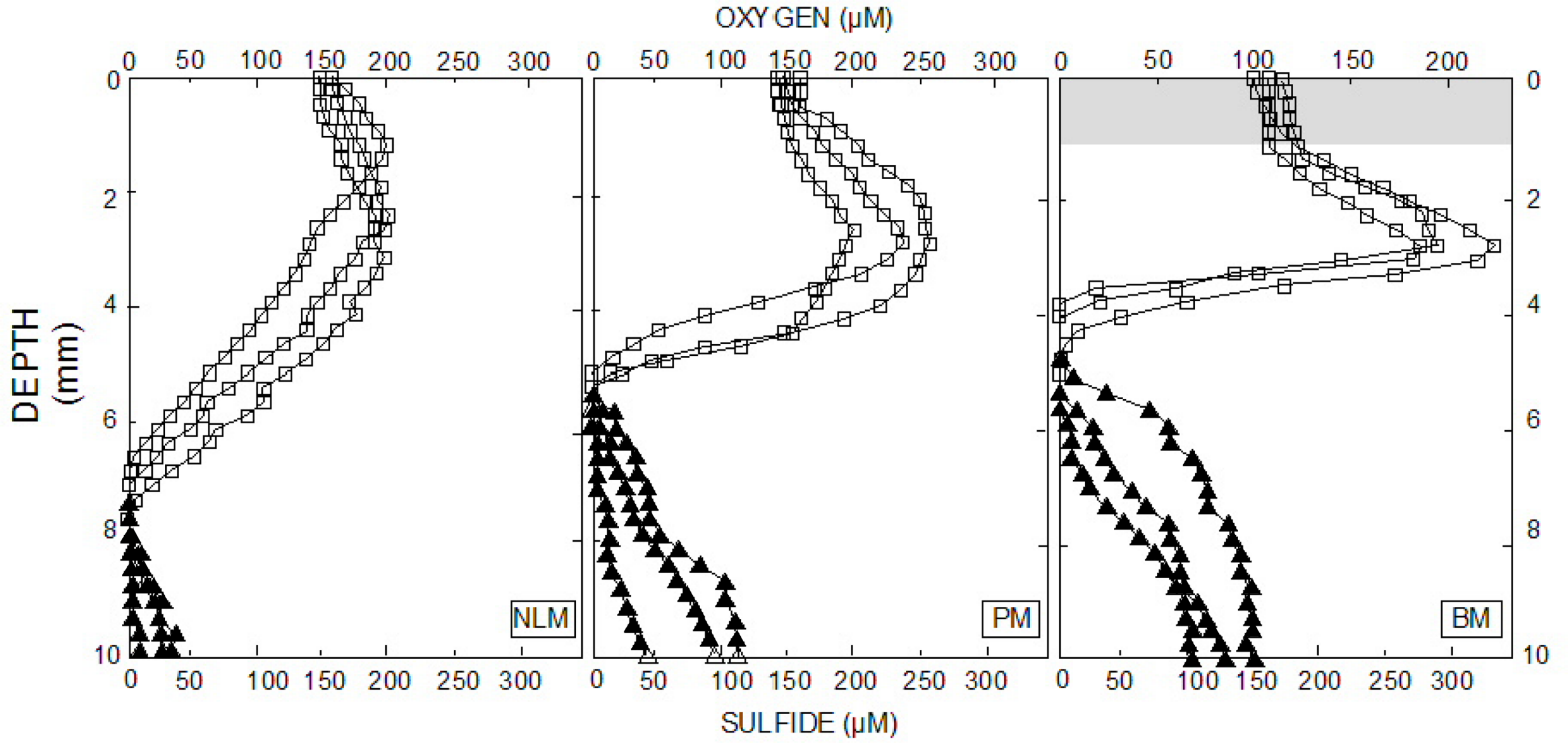
In situ depth profiles of oxygen and sulfide. Microelectrode measurements were obtained during peak photosynthesis (12:00–14:00) Individual profiles of O_2_ (squares) and sulfide (triangles) shown. The O_2_ peaks and maximum values of sulfide that were observed were higher in microbialites than in the non-lithifying mat. The O_2_ penetration was considerably deeper in the mat compared to the microbialites, indicating higher rates of O_2_ production and consumption in the latter.

During peak photosynthesis (12:00–14:00), the distribution of O_2_ and sulfide with depth revealed differences between the mat and microbialites (Fig 2). The light intensity of PAR was 1,410–1,620 μE.m^-2^.s^-1^ at the surface of NLM, and 1,850–2,550 μE.m^-2^.s^-1^ at the surface of PM and BM. High oxygen maxima (~130% of O_2_ saturation observed between 1.75 and 3.5 mm depth in NLM and 120–170% of O_2_ saturation at 2.75–3 mm depth in PM) coupled with a relatively deep O_2_ penetration, and free sulfide at depth in NLM and BM suggested these are dynamic sediment systems. The profiles in PM showed a rapid increase of O_2_ (>200% of O_2_ saturation) followed by a steep decline anoxic conditions at 4 mm. Build up of sulfide to 100–150 μM indicated that the pink microbialites were the most active of the three systems.

Polysulfides were present in all mat and microbialite samples, with the highest values in NLM, followed by PM and BM (S1 File). Concentrations measured at the end of the night (i.e., anoxic period) were considerably higher than during the daytime. Values determined in non-lithifying mats (MA1; [35]) in Tebenquiche were 1.5–2.3 times higher than in La Brava. Thiosulfate concentrations followed a similar pattern, peaking in NLM of Brava at 33 μM in the day and 112 μM, at the end of the night. Daytime values for the Tebenquiche non-lithifying mat MA1 were 2–2.5 times higher than for NLM. Nighttime values for MA1 were not measured.

Analyses showed carbonates and halite as the only mineral phases (S1 Table). The rhizome-associated concretion (RAC) was located at the edge of an extensive carbonate bank. In the bulk analyses of the four different systems, halite constituted a major part of the minerals, presumably partially a porewater drying artifact originating from the organic portions of the mat. The halite amount decreased as the organic content (predominantly extracellular polymeric substances (EPS)) decreased, and as the mat types became more lithified, and their aragonite content increased. Organic content in g/g dry sediment was measured as: NLM 0.345, BM 0.257 and RAC 0.163. The PM organic content was not measured. The NLM contained the least amount of aragonite followed by the microbialites, PM and BM, and the RAC.

In the detailed analyses of individual layers, the NLM top layer sample showed aragonite, traces of gypsum, and the remainder halite. In the second layer, aragonite and halite were still present. The third layer did not contain aragonite, but some halite was measured. The three individual layers of the BM microbialite all contained aragonite as major component.

### Sediment communities

The highest diversity indices and equitability were found in PM, which had the lowest dominance of OTUs (Table 2). The diversity indices and equitability decreased in the following order: BM>NLM>RAC. When the diversity per layer was investigated in two selected systems (NLM and BM), a general trend of increasing diversity indices with depth was found (S2 Table).

**Table 2:** Diversity metrics for the total sampled environments, using 16S rDNA V4 region sequences clustered at similarity level of 0.97 and normalized to 1,800 sequences per sample.

Description	Seqs/Sample	Chao1	Dominance	Equitability	Observed species	Shannon	Simpson
NLM	1800	486	0.03	0.79	317	6.58	0.97
PM	1800	514	0.01	0.86	363	7.33	0.99
BM	1800	358	0.02	0.85	273	6.91	0.98
RAC	1800	416	0.05	0.76	275	6.15	0.95

**Fig 3:**
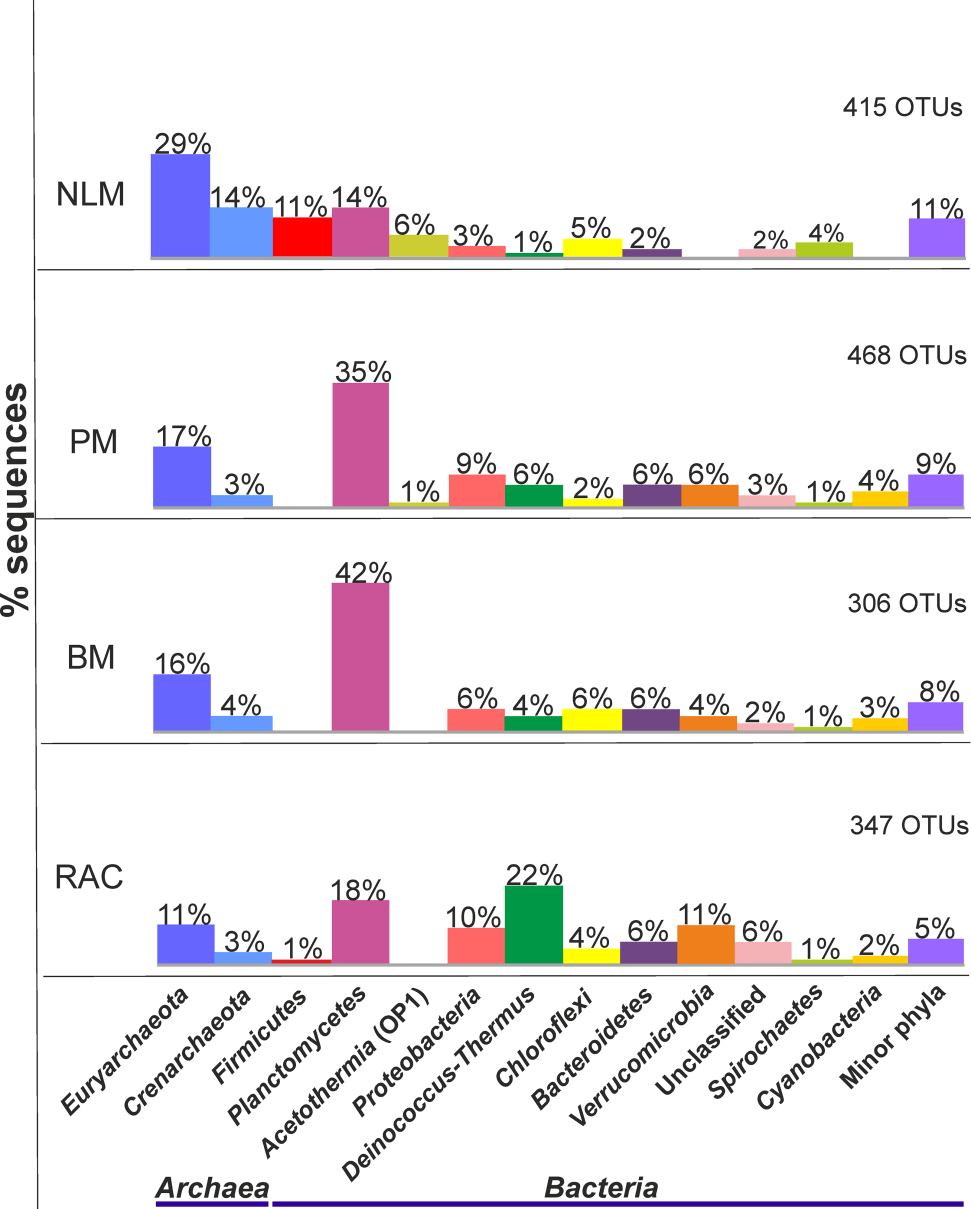
Comparison of prokaryotic diversity by sampling location. Bulk samples based on 16S rRNA gene sequences of the V4 hypervariable region. Bars indicate the contribution of each phylum to the total diversity. Phyla representing less than 1% of the total diversity are grouped as “minor phyla”.

Mat sample NLM showed the highest dominance of *Archaea* (43% of total diversity), with 29% *Euryarchaeota* and 14% *Crenarchaeota* (Fig 3). *Bacteria* accounted for 57% of total diversity in NLM, with *Planctomycetes* (14%), *Firmicutes* (11%) and *Acetothermia* (6%) as the most abundant taxa. The diversity in PM was dominated by the phyla *Planctomycetes* (35% of total diversity), followed by *Euryarchaeota* (17%) and *Proteobacteria* (9%) (Fig 3). The prokaryotic diversity in BM was represented by the phyla *Planctomycetes* (42% of total diversity), followed by *Euryarchaeota* (16%). The diversity in RAC was the lowest of the La Brava ecosystems analysed and was dominated by *Deinococcus*-*Thermus* (22%) and *Planctomycetes* (18%). Phyla representing less than 1% were grouped as “minor phyla” in the bulk analyses.

**Fig 4:**
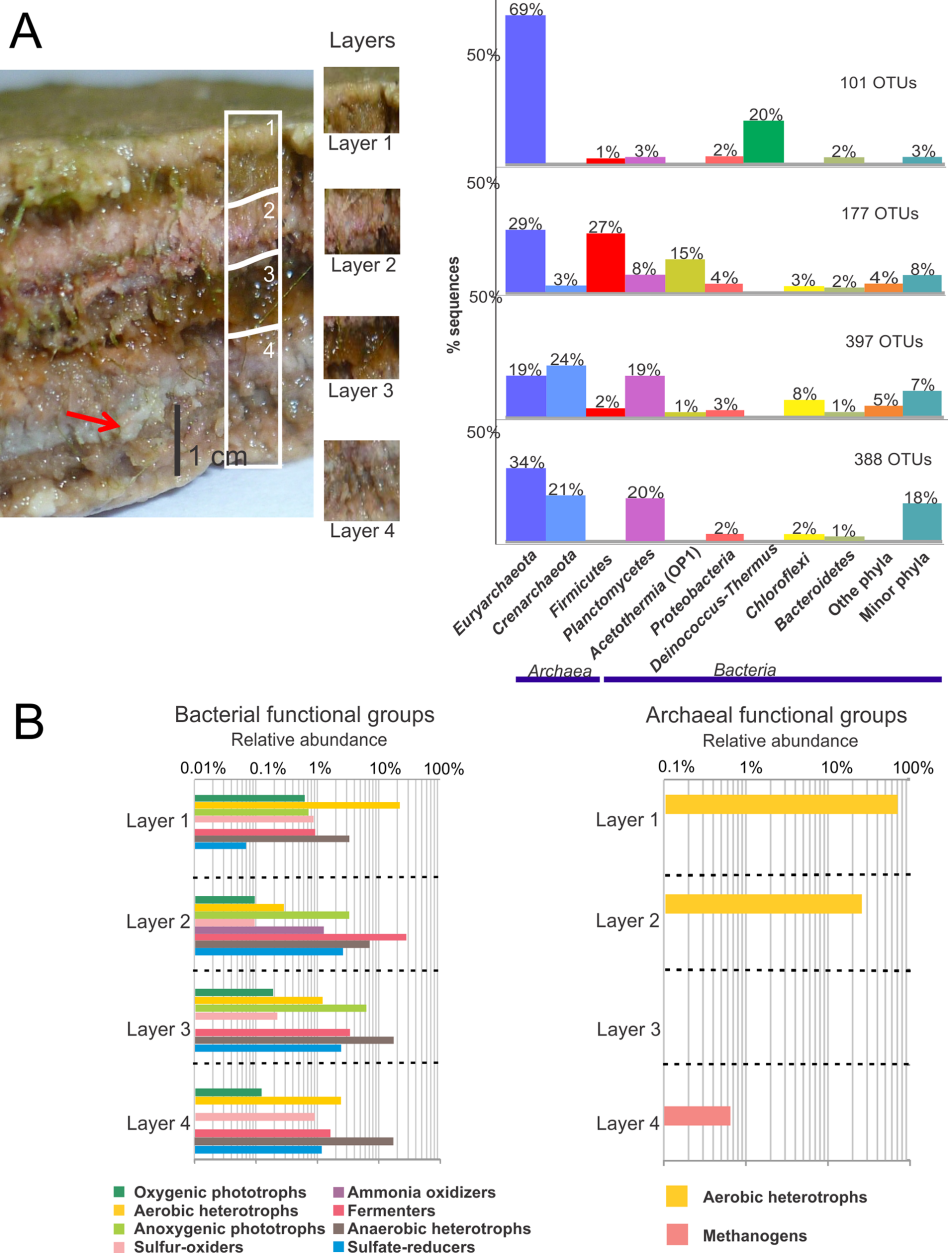
Prokaryotic diversity by layer in NLM. **(**A) Relative abundance of the phyla based on bacterial 16S rRNA gene sequences of the V4 hypervariable region. Phyla that represent less than 1% of total diversity are grouped in “minor phyla”. (B) Functional diversity abundance by layer. Percentage of sequences belonging to Bacteria and Archaea represented layer per layer. Notice the log scale. Functional groups were inferred from literature search of the metabolic capabilities of each classified microorganism present in the sample.

**Fig 5:**
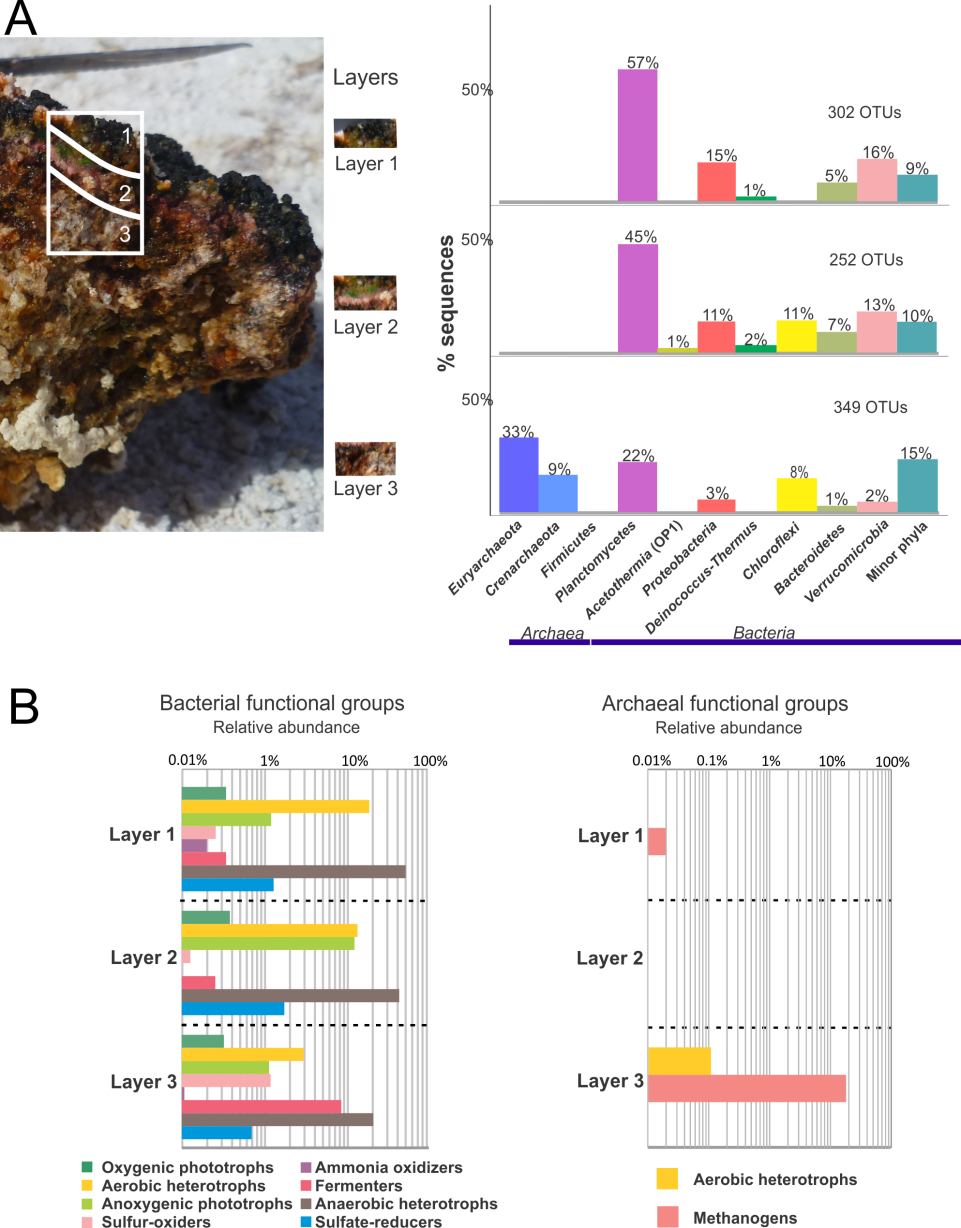
Prokaryotic diversity by layer in BM. **(**A) Relative abundance of the phyla based on bacterial 16S rRNA gene sequences of the V4 hypervariable region. Phyla that represent less than 1% of total diversity are grouped in “minor phyla”. **(**B) Functional diversity abundance by layer. Percentage of sequences belonging to Bacteria and Archaea represented layer per layer. Notice the log scale. Functional groups were inferred from literature search of the metabolic capabilities of each classified microorganism present in the sample.

Detailed functional group analyses in consecutive depth horizons were carried out for NLM (Fig 4, S2 Fig) and BM (Fig 5, S3 Fig). In NLM, probable bacterial functional groups including oxygenic phototrophs, aerobic heterotrophs, anoxygenic phototrophs, sulfur oxidizers, fermenters and anaerobic heterotrophs were present at all layer depths. Ammonia oxidizers were only observed in Layer 3 where the dominant functional group appeared to be fermenters. Anaerobic heterotrophs, including sulfate reducers, were the dominant groups in Layers 3 and 4. Archaeal functional group analysis showed aerobic heterotrophs dominating the first and second layers. Methanogens were detected only in the deepest layer (4). The Crenarchea were represented by MBGB, which were present in all layers and decreased with depth; however, their probable functional role is unknown. In BM, the bacterial functional groups corresponding to oxygenic phototrophs, aerobic heterotrophs, anoxygenic phototrophs, sulfur oxidizers, fermenters, anaerobic heterotrophs and sulfate reducers were distributed throughout the layers. The surface (Layer 1) was dominated by aerobic heterotrophs and anaerobic heterotrophs. Cyanobacteria were present in Layer 1 but relatively low in numbers. Archaeal functional groups included aerobic heterotrophs, and methanogens. The third layer was dominated by methanogens.

**Fig 6:**
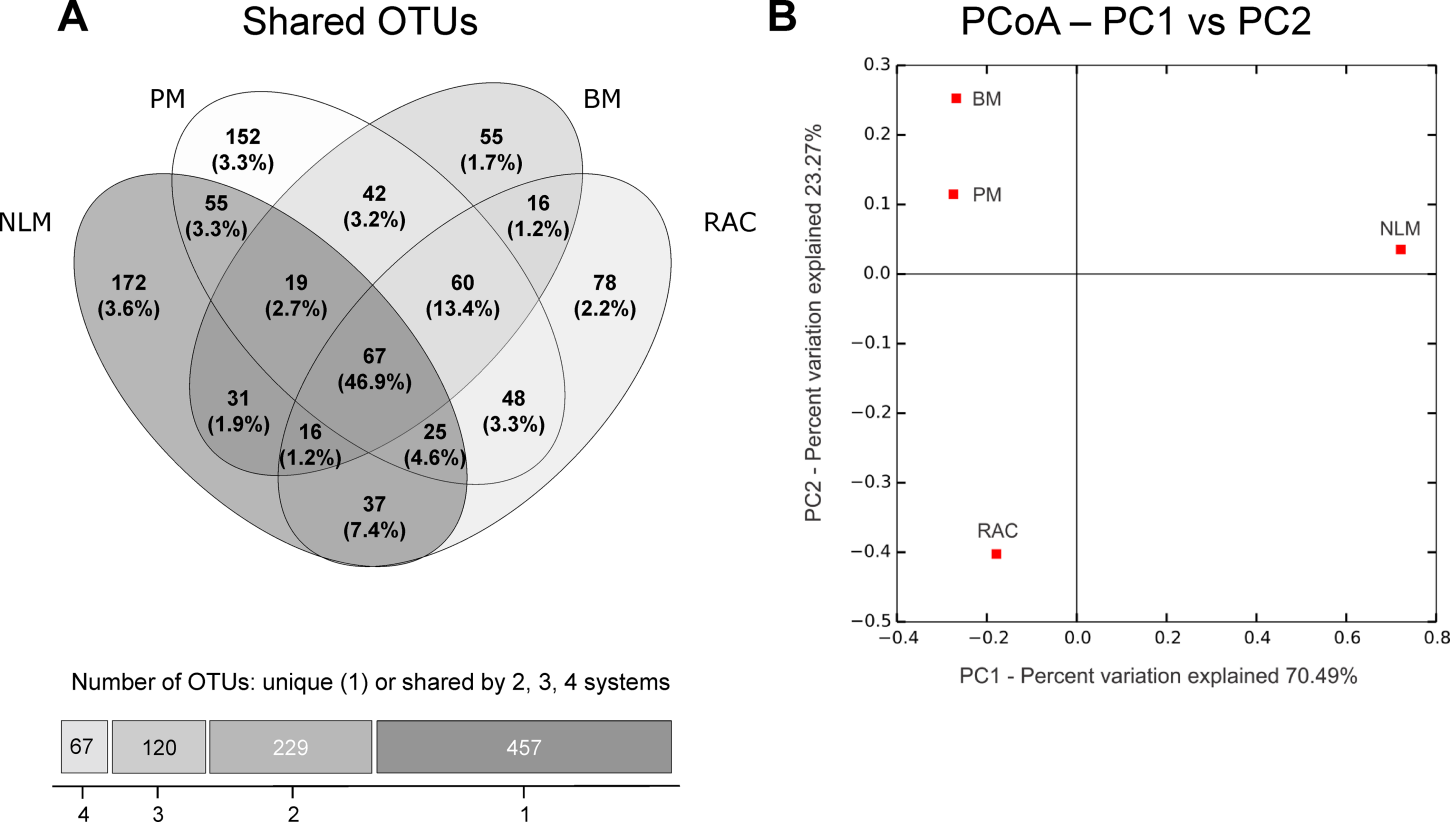
OTU-level comparison of the sites using 97%-sequence identity. (A) Venn diagram showing the number of OTUs shared between the sites, the percentages in parentheses represent relative abundance of sequences assigned to the OTUs. The bar graph shows the distribution of shared OTUs: from left to right: 67 OTUs are shared by all four systems, 120 OTUs are shared by three, 229 are shared by two and 457 OTUs are unique (not shared with other systems). (B). Principal Coordinates Analysis (PCoA) based on OTUs in which 94% of the variation is explained by the first two axes.

Similarities of the microbial ecosystems seen at macroscopic level were supported by OTU analysis, *i*.*e*., the two microbialites are more closely related to each other than to the other systems. This can be clearly seen in the Principal Coordinates analysis based on phylogenetic Unifrac weighted distances between the samples with OTUs at 97% identity (Fig 6B). These distances account for the differences both in OTUs and their abundances. Based on this, the samples are separated in three groups: i) NLM (mat); ii) PM and BM (microbialites); and iii) RAC. The distribution of OTUs (Fig 6A) shows that about half of the sequences are common to all samples (67 OTUs comprising 46.9% of the sequences). The core microbiome included members of *Deinococcus*-*Thermus*, *Crenarchaeota*, *Euryarcheota*, *Planctomycetes*, *Gammaproteobacteria and Verrucomicrobia*. Interestingly, only one OTU from each *Alphaproteobacteria*, *Deltaproteobacteria*, and *Cyanobacteria* were among these common OTUs, with abundances below 0.5%. Core OTUs were unevenly distributed (S3 Table). For example, OTU1172, affiliated to family *Trueperaceae*, phylum *Deinococcus*-*Thermus*, comprises 18% of the sequences in RAC, but less than 3% in the other systems. Another example, OTU1110, affiliated to class *MBGB*, phylum *Crenarchaeota*, represents 10% of the sequences in NLM, and less than 2% in the other systems.

### Multivariate analysis

**Fig 7:**
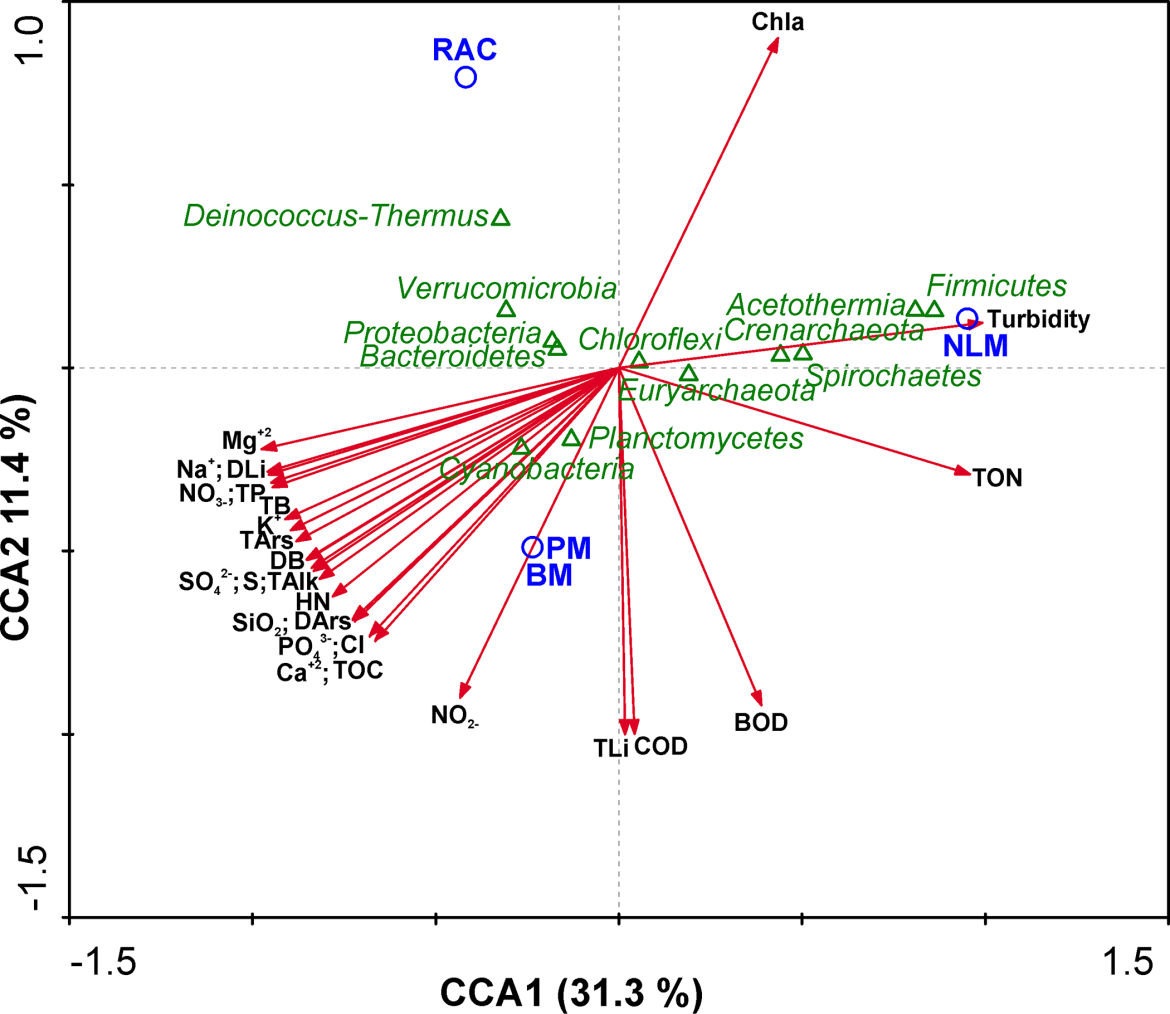
Canonical Correspondence Analysis (CCA) of the prokaryotic lineages, sampling sites and environmental properties. Triangles represent response variables (OTU abundances). Arrows represent quantitative explanatory variables (physico-chemical parameters) with arrowheads indicating their direction of increase. Circles represent qualitative explanatory variables (sites). BOD: Biochemical oxygen demand; COD: Chemical oxygen demand; Chla: Chlorophyll a; HN: Hardness; TAlk: Total alkalinity; TOC: Total organic Carbon; NO-3: Nitrate; NO-2: Nitrite; TON: Total organic nitrogen; TP: Total phosphorus; PO43-: Phosphate; SO42-: Sulfate; S: Sulfur; Na+: Sodium; Cl-: Chloride; K+: Potassium; Mg2+: Magnesium; Ca2+: Calcium; DB: Dissolved boron; TB: Total boron; DLi: Dissolved lithium; TLi: Total lithium; SiO2: Silica; DAr: Dissolved Arsenic; TAr: Total Arsenic.

The possible relationship between the prokaryotic community composition and environmental variables was examined by canonical correspondence analysis (CCA) (Fig 7). CCA axes 1 and 2 explained 42.7% total variance data. The lowest conductivity was found in the waters near the NLM compared to the other samples. This was seen in the correlation of the NLM with major ions sodium (Na+), chloride (Cl-), potassium (K+) and magnesium (Mg2+). Turbidity was much higher at the NLM site than the areas where microbialites (PM and BM) were forming. The NLM site was the only (eco) system in which the phyla *Firmicutes* and *Acetothermia* were present. Besides a lower turbidity, the sites where microbialites and the rhizome-associated concretion formed coincided with the highest orthophosphate (PO43-) and silica (SiO2) concentrations. The phyla more positively influenced by these conditions appeared to be *Deinococcus*-*Thermus*, *Verrucomicrobia* and *Cyanobacteria*. These phyla were absent or comprised only a small fraction of total diversity in NLM.

## Discussion

### Taxonomic and functional composition of the sediment communities

The most significant finding of the present study was the large proportion of archaeal diversity that included *Euryarchaeota* and *Crenarchaeota* together with a large proportion of bacterial phyla like *Firmicutes*, *Planctomycetes* and *Acetothermia*. Previous studies from an earlier campaign (March 2012) at Laguna La Brava looking at only bacterial diversity, showed that *Bacteroidetes* and *Proteobacteria* constituted major phyla in the bacterial diversity of microbial mats and microbialites [32]. In the current study, members of the phyla *Crenarchaeota* and *Euryarchaeota* were the most abundant in non-lithifying mats (NLM) followed by species belonging to *Firmicutes*, *Acetothermia*, and *Planctomycetes*. In contrast, both microbialite systems, PM and BM, were dominated by *Planctomycetes* with a lower proportion and affiliation of *Euryarchaeota*: *Methanomicrobia* and *Thermoplasmatales* in the microbialites, in contrast to *Halobacteria* in the mat (Figs 4 and 5, S3 Table). These lineages have not been reported before as dominating mats or microbialites. These results are only based on 16S rRNA gene amplicon sequencing with F515 and R806 primers [49]. However, more recently these primers have been showed to be biased against *Crenarchaeota* and *Thaumarchaeota* [57]. Given this bias, the relative abundance of these phyla might be underestimated.

A characteristic of microbial mats is the presence of cyanobacteria and other phototrophs [58]. In this study cyanobacteria were found in low abundance (2–4%) in the microbialites, and were below 1% in NLM. The microbial diversity of the endolithic community of halite crusts in the Atacama Desert, similar to those surrounding La Brava, was composed of about 6% cyanobacteria [59]. Although chlorophyll a was found in the water column of the lake, previous investigations of the La Brava mats and microbialites, showed Chl*a* levels were below detection by high performance liquid chromatography (HPLC) [32]. Such reduced Chl*a* levels can be attributed to high levels of UV, as was previously reported for Antarctic benthic cyanobacteria [60].

Subsurface oxygen maxima, usually found in microbial mats [14, 45, 58] were evident in the NLM, PM and BM. However, compared with typical mat systems their maximum values were much lower (approximately 120–200% here vs. >400% in typical mats) [6, 61]. This oxygen could have been produced by diatoms (seen in SEM, S1 Fig) or by a few cyanobacterial phyla present in low diversity but in higher numbers but with a low specific Chl*a* content.

Anoxygenic phototrophic sequences are also not well-represented in NLM and microbialites from La Brava, despite the presence of sulfide near the surface of the mats and microbialites (Fig 2). This finding is in contrast with similar high altitude systems like Socompa [29], Llamara [34], and Tebenquiche [35], and also geographically different microbial mats or microbialites systems like Shark Bay [62, 63] or Guerrero Negro [64]. Chloroflexus-type sequences (green non-sulfur bacteria) were not very common in La Brava even though they are generally abundant in photosynthetic mats [64–69].

The low abundance of phototrophs implies that in the La Brava systems, particularly in NLM, carbon is fixed using alternative pathways. Perhaps little known and relatively uncharacterized phylogenetic groups, such as the *Planctomycetes*, *Firmicutes*, *Acetothermia*, *Euryarchaeota* and *Crenarchaeota*, are fulfilling a significant role. A genome reconstructed from a fosmid library of *Candidatus* ‘Acetothermum autotrophicum’ revealed genes encoding for the folate-dependent Wood-Ljungdahl (acetyl-CoA) pathway of CO_2_ fixation [70]. *Acetothermia*, extremophiles first found in Obsidian Pool (Yellowstone NP), are currently uncultivated, but have been identified in a variety of environments using culture-independent methods [70–75]. Metagenomic analysis of modern stromatolites in Socompa, Andes, Argentina revealed that alternative modes of CO_2_ fixation, such as the acetyl-CoA pathway and the reverse TCA cycle, were present in these microbialites [37]. The presence of genes for CO_2_ fixation in *Acetothermia*, which were present in NLM and only in minor amounts in microbialites of this study, suggests they could contribute to primary production through chemolithoautotrophic acetogenesis. Diatoms were observed by electron microscopy only at the surface of some mats (S1 Fig), and may also contribute to CO_2_ fixation.

The unusually high presence of archaea in this study will require further investigation. The most abundant euryarchaeal OTUs were classified into the deep-sea hydrothermal vent euryarcheal group 1 (DHVEG-1), members of which were present in the water column and sediment of anoxic deep-sea hydrothermal vents [76]. DHVEG-1 was also found in deeper, anoxic layers of an evaporitic microbial mat in Kiribati [77]. A major fraction of the *Crenarchaeota* in La Brava is made up by Marine Benthic Group B (MBGB). This crenarchaeal group is abundant, diverse and widespread in marine sediments [78] including Black Sea mats surrounding cold seeps [79]. MBGB are often associated with sulfate-reducing bacteria in sulfate-methane transition zones [80]. Although there is no definite evidence for involvement of these *Crenarchaeota* in anaerobic oxidation of methane, their role has been proposed [78, 79, 81]. The role of MBGB in these Atacama Desert systems is not known.

### Influence of water chemistry in community composition

The diversity in microbial ecosystems investigated here was compared to their overlying water chemistry using CCA analysis. This analysis showed three groupings; NLM was distinctly different from BM and PM, and all were different from RAC (Fig 7). This distribution was also supported by unifrac distance based PCoA (Fig 6B). The major differences seemed to be due to turbidity and salinity, but the CCA axes explained less than 43% of total differences indicating that other aspects of the overlying water chemistry may contribute to the microbial populations, especially high levels of metals such as lithium, arsenic, magnesium, and calcium (Table 1).

Increased turbidity suggests reduced light levels, and a less pronounced oxygen peak was observed in NLM compared to PM and BM (Fig 2). This could favor growth of anaerobes in NLM, such as representatives from *Firmicutes* and *Acetothermia*. The bulk molecular data showed that NLM also had a higher diversity (415 OTUs) than the most lithified of the microbialites (BM, 306 OTUs). This is consistent with results from mats in a hypersaline lake in the Bahamas where the non-lithifying mats supported a higher total diversity than the lithifying mats [82, 83]. One of the characteristics of the mats in the Tebenquiche and La Brava systems is high levels of metals such as lithium, arsenic, magnesium, and calcium [32, 35]. High levels of metals may require a physiological response to cope with toxicity. The phosphate concentration in the water at the site of microbialites was about 25% higher than in the water overlying NLM (Table 1) corresponding with 20–25% higher water column concentrations of some metals (As, Mg, Ca) (Table 1). Phosphate can be assimilated and stored as inorganic polyphosphate (polyP) by many microbes [84]. In addition, polyP play a fundamental role in metal resistance in bacteria [85] and fungi [86], possibly chelating cations [84, 87]. Representatives of the phyla *Deinococcus*-*Thermus*[88], and *Verrucomicrobia* [89] inhabiting the microbialites and rhizome-associated concretions may be using polyP in metal resistance.

### System comparison by layer

A defining characteristic of the different ecosystems in this study is the degree of carbonate mineral precipitation. This mineral precipitation occurs at different depth horizons and is likely the result of changes to the pore water chemistry caused by the interaction of different microbial metabolisms [11, 14]. *Archaea* were present in all four analyzed layers of NLM (Fig 4), where they represented the main phylogenetic groups. Among *Euryarchaeota*, aerobic heterotrophs belonging to *Halobacterium* dominated the upper layers (Fig 4). *Methanomicrobia* were detected in the lower three layers of NLM and the deepest layer of BM, where they are likely involved in methane production. Methane production and sulfate reduction are metabolisms favoring carbonate precipitation [11, 90] and could explain the presence of deep lithified layers (e.g., deepest layer in NLM).

The analysis by layer indicated the presence of *Cyanobacteria* in all layers from NLM and BM, though in exponentially fewer numbers than other phyla. These are oxygenic phototrophs and are more commonly the main autotrophs in mats. They also produce exopolymeric substances and fix nitrogen [82, 91]. Oxygen production in the first millimeters of the mat/microbialite (Fig 2) suggests that they would be performing photosynthesis in the first layer. Cyanobacteria present in all layers have also been observed in other mat systems [29, 36, 62, 91, 92]. Oxygenic photosynthesis is proposed to promote carbonate precipitation in mat systems by increasing pH, but the low abundance of cyanobacteria here makes that an unlikely driving mechanism. Other possible autotrophic metabolisms in these systems, discussed above, may be the drivers of primary productivity in these mats. The role of alternative autotrophic metabolisms in mineral precipitation is less well known.

Heterotrophs were present in all the layers of both NLM and BM; and included aerobes (*Bacteroidetes*, *Planctomycetes*, *Deinococcus-Thermus*, *Alphaproteobacteria*, *Betaproteobacteria* and *Gammaproteobacteria*), and anaerobes (*Verrucomicrobia*, *Bacteroidetes* and *Planctomycetes*). *Bacteroidetes* could have an ecological role of breaking down macromolecules, including EPS [63, 93]. EPS degradation is assumed to be a critical step in carbonate precipitation [14], and might explain carbonate minerals in Layer 4 of NLM.

Anoxygenic phototrophs (e.g., *Alphaproteobacteria*, *Gammaproteobacteria*, and *Chloroflexi*) were present in the upper three layers of both NLM and all layers of BM, but in low abundance. Anoxygenic phototrophic metabolisms precipitate carbonate minerals [90]. Their distribution coincides with fermenters (*Clostridia*, *Chloroflexi* and *Spirochaetes*), which were present in all the layers, but better represented in the soft mats than in lithified systems. Lower oxygen production in NLM supports development of fermenters, the metabolism of which dissolves carbonates [90].

Sulfate reducers were affiliated to *Deltaproteobacteria*, and mostly present in the bottom three layers in NLM, however they were found in all of the layers, including the surface, of BM.Their relative abundances were 2.5% of Deltaproteobacteria in NLM and 0.8% in BM (S3 Table). In NLM layers, *Desulfurellales* order was more abundant in layers 2 and 3, while *Syntrophobacterales*, *Desulfobacteraceae* family, dominated layer 4 (S2 Fig). In BM, *Syntrophobacterales*, *Syntrophaceae* family dominated layer 1, while *NB1-j* order was prevalent in layer 2 (S3 Fig). Sulfate reduction has been found in the oxic zones of hypersaline microbial mats in Guerrero Negro [94], Solar Lake [95], Kiritimati Atoll [96], the Bahamas [2, 82], and Shark Bay [97]. Sulfate reducer metabolism may contribute to net carbonate precipitation [11, 90]. This is consistent with their presence in the top layer of microbialites in La Brava.

Sulfur oxidizers were overall scarce, with less than 0.2% relative abundance. They were represented by the *Gammaproteobacteria Thiomicrospira*, found in all layers in the studied systems, but more abundant in the bottom layers (Figs 4 and 5, S3 Table).*Thiomicrospira* spp. are often found in mats, where they are capable of sulfide and thiosulfate oxidation using either oxygen or nitrate [98]. Their role in carbonate dissolution is well known in these systems, and it is the balance of mineral precipitating and mineral dissolution processes that leads to net precipitation, potentially resulting in lithification [90, 99]. Besides *Thiomicrospira* it must be noticed that other more abundant organisms can be contributors to sulfur oxidation, such as anoxygenic photosynthesizers that use sulfide to feed the electron chain, but those were not included in the “sulfur oxidizers” group in the figure.

### Comparison of laguna La Brava to nearby laguna Tebenquiche

La Brava and Tebenquiche systems possess unusual diversity compared to known mats and microbialite systems [58, 64, 99–105], since they contain a large proportion of *Euryarcheota* and *Chrenarcheota*, and a very low proportion of *Cyanobacteria* (S4 Fig). Both systems were sampled in November 2012, and the results from Tebenquiche campaign are reported elsewhere [35]. Of the bacteria present, *Planctomycetes* dominate in La Brava, while in Tebenquiche bacterial diversity includes groups like *Firmicutes*, *Acetothermia*, *Chloroflexi* and *Planctomycetes*. The distribution of overall diversity is also different between the two systems. In La Brava, diversity increases with depth while this does not occur in Tebenquiche.

In La Brava carbonate precipitation is prevalent while Tebenquiche is a gypsum precipitating system. The influence of the local bacterial communities in these processes is unclear, but since sulfate reducer metabolism may contribute to net carbonate precipitation [11, 90], this is consistent with *Deltaproteobacteria* being more abundant in La Brava systems, particularly in NLM and PM. Despite *Deltaproteobacteria* being less abundant, sulfur metabolism is likely more active in Tebenquiche. Concentrations of all measured sulfur forms, including sulfate, sulfide, thiosulfate and polysulfide, are higher in Tebenquiche. Anoxyphototrophs have a potentially important role in CO_2_ fixation coupled to sulfide oxidation in both systems. In both La Brava and Tebenquiche, this group comprises purple-nonsulfur bacteria, some *Chloroflexi* and *Halorhodospira* (*Chromatiales*). Their abundances varies between 0.1 to 5%, with the highest values in La Brava mats. *Chloroflexi* are typically dominant in mats, but perhaps the high UV or unusual ionic composition of the water restricts these green filamentous sulfur bacteria in both Atacama ecosystems, being only abundant in the RAC1 system in Tebenquiche (S4 Fig). Perhaps in Tebenquiche mats (MA1 and MA2), the higher proportion of Archaea is contributing to these and other metabolisms, as only a fraction of them have known functions.

## Conclusions

The extreme conditions in the La Brava system select for unusual diversity where *Archaea*, *Acetothermia*, *Firmicutes* and *Planctomycetes* may play fundamental roles, and net mineral precipitation may arise from a combination of unique metabolisms not previously seen. For example, lineages like *Acetothermia* [70] or *Crenarchaeota* [106] might contribute the majority of primary production. Higher turbidity and a lower salinity in NLM *vs* BM promoted different microbial communities, lower subsurface oxygen production, and less mineral precipitation. *Cyanobacteria* have been studied extensively in other systems for their ability to promote mineral precipitation by increasing porewater pH, but they are present here in very low abundance, and are not likely to contribute significantly to mat primary productivity or pH changes in the porewater. Furthermore, diatoms were only present at the surface of some mats, and may have contributed to CO_2_ fixation in the mats, but not to the oxygen peak which was always at the subsurface. Sulfate-reducing, fermenting and sulfur-oxidizing roles are filled by bacteria and could have archaeal contributors, and in many cases phyla have been identified that contain genes for unusual non-oxygen dependent lifestyles, both autotrophic and heterotrophic. For example, MBGB have been found here in relatively high abundance. They have previously been identified in sulfate methane transition zones in environments like/sediments of/ the Black Sea, Santa Barbara Basin [80], and hydrate seeps off the coast of Oregon [79] but their role is unknown and their presence here warrants further study.

Anoxic conditions and high UV radiation on early Earth may have required similar metabolic strategies to those operating in these high altitude lakes, making this habitat an opportunity to study primitive biogeochemical cycles. Ongoing scientific research is needed, and will form the basis to justify environmental protection of this unique microbial system. The insights this research provides may improve understanding of life on early Earth.

## Supporting information

S1 Fig Scanning electron micrographs of diatoms. Surface of BM (Panel A) and NLM (Panel B).
(TIF)

S2 Fig Bacterial functional diversity abundance on each layer from NLM. Diversity is disclosed at several taxonomic levels. Each group is displayed separately: oxygenic phototrophs, anoxygenic phototrophs, aerobic heterotrophs, anaerobic heterotrophs, fermenters, and sulfate reducers.
(TIF)

S3 Fig Bacterial functional diversity abundance on each layer from BM. Diversity is disclosed at several taxonomic levels. Each group is displayed separately: oxygenic phototrophs, anoxygenic phototrophs, aerobic heterotrophs, anaerobic heterotrophs, fermenters, and sulfate reducers.
(TIF)

S4 Fig Taxonomic profiles at phylum level of La Brava and Tebenquiche samples. Samples from different campaigns are shown.
(TIF)

S1 File Porewater polysulfides and thiosulfate comparison between LaBrava and Tebenquiche. (XLSX)

S1 Table Mineral content of bulk and layer samples. (XLSX)

S2 Table Diversity metrics for the layers belonging to samples NLM and BM, using 16S rDNA V4 region sequences clustered at similarity level of 0.97 and normalized to 1,800 sequences per sample. (XLSX)

S3 Table OTU table and taxonomic assignment for bulk samples. (XLSX)
