# Supplementary figures and images for: Prokaryotic diversity and biogeochemical characteristics of benthic microbial ecosystems at La Brava, a hypersaline lake at Salar de Atacama, Chile
Source: PLoS One. 2017 Nov 15;12(11):e0186867. doi: 10.1371/journal.pone.0186867 (PMC5687714; doi:10.1371/journal.pone.0186867)

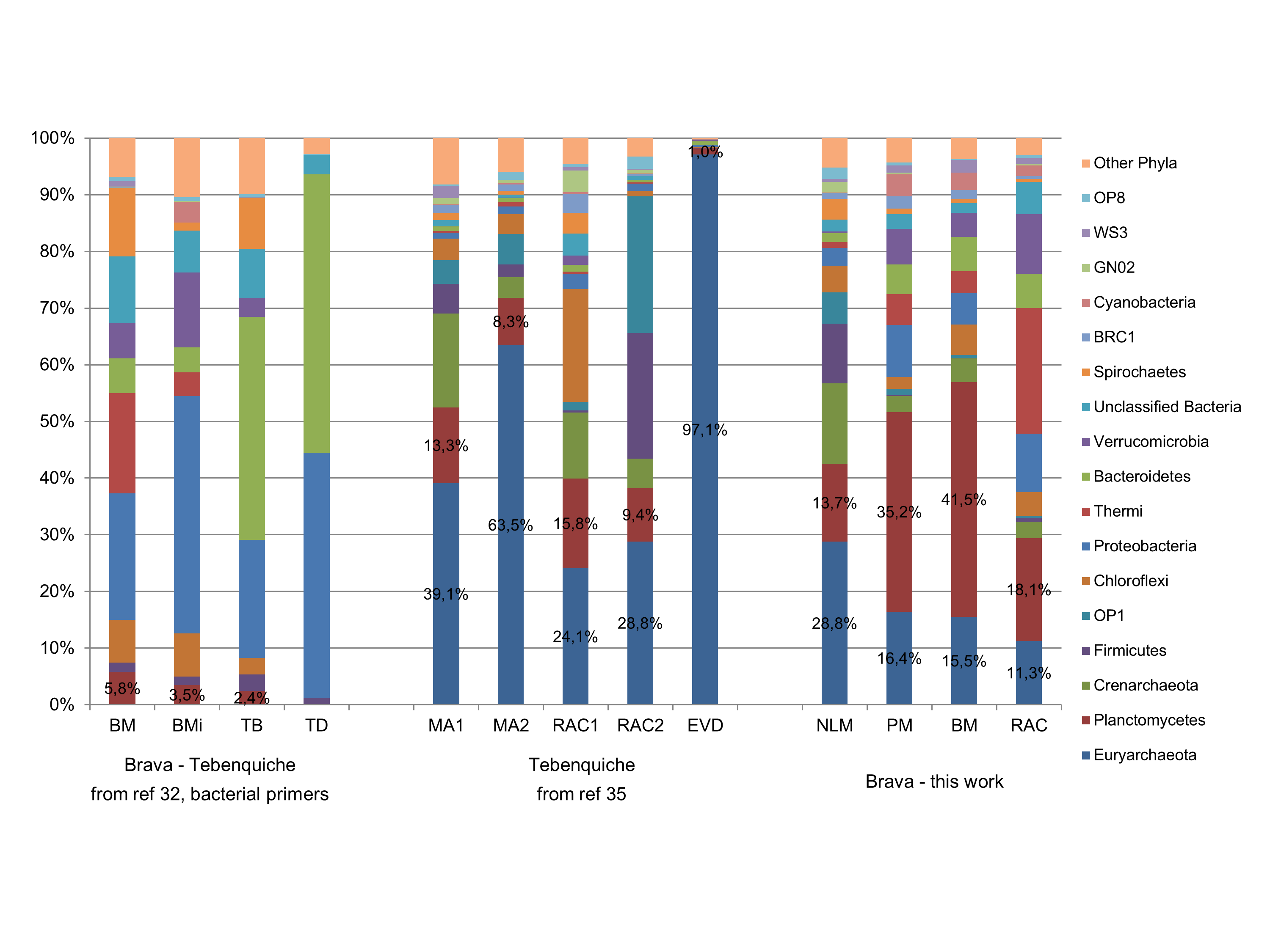

Supplement: S4 Fig — Samples from different campaigns are shown. (TIF) [file pone.0186867.s004.tif]
